# A rare case of 2q37 deletion syndrome presented with patent foramen ovale

**DOI:** 10.1002/ccr3.6970

**Published:** 2023-11-19

**Authors:** Ahmed Zaki, Nour Shaheen, Mohamed Hosny, Abdelraouf Ramadan, Abdulqadir J. Nashwan

**Affiliations:** ^1^ Faculty of Medicine Cairo University Cairo Egypt; ^2^ Faculty of Medicine Alexandria University Alexandria Egypt; ^3^ Louisville University Louisville Kentucky USA; ^4^ Faculty of Medicine Helwan University Cairo Egypt; ^5^ Hamad Medical Corporation Doha Qatar

**Keywords:** 2q37 deletion syndrome, cardiac anomalies, chromosome 2, comparative genomic hybridization, patent foramen ovale, single nucleotide polymorphism

## Abstract

This case report presents a 3‐year‐old female child diagnosed with 2q37 deletion syndrome and patent foramen ovale, and the improvement in hypotonia and gross motor delay after 1 year of physical therapy. This case highlights the importance of thorough examination and diagnostic testing in identifying underlying causes of developmental delays.

## Graphical abstract

In this case of 2q37 deletion syndrome and PFO, we highlight the effectiveness of physical therapy in managing such cases. Both thorough clinical examination and proper diagnostic tests are essential.

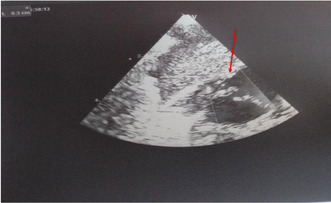

## 1 INTRODUCTION

2q37 deletion syndrome is a genetic disorder characterized by the loss of a small portion of genetic material located on chromosome 2. The specific deletion occurs within the 2q37 region, which is located on the long q arm of chromosome 2 and is divided into three cytogenic areas containing a total of 179 genes. However, only 11 of these genes have been identified as being related to the 2q37 deletion syndrome. The amount of genetic material lost in this disorder can vary from individual to individual, and it is missing in one of the patient's two copies of chromosome 2.^1^ The majority of individuals diagnosed with 2q37 microdeletion syndrome are considered to be isolated cases, meaning that they do not have a familial history of the disorder. Additionally, patients with this disorder typically exhibit a normal parental phenotype karyotype, meaning that there are no observable chromosomal abnormalities in their parents.^2^ Patients diagnosed with 2q37 microdeletion syndrome may experience a range of developmental effects, which can vary greatly from individual to individual. Common symptoms in infants may include low muscle tone and feeding difficulties. Developmental delay, learning disabilities, and physical abnormalities such as brachycephaly, obesity, and digit abnormalities are also commonly observed. Some patients may also experience respiratory issues such as asthma, ear and chest infections, seizures, and autism. Facial characteristics can also vary among patients, but may include a prominent forehead, sparse flared medial eyebrows, depressed nasal bridge, V‐shaped nasal tip, high‐arched palate, alopecia totalis, boxy skull with prominent forehead, and up‐slanting palpebrae.^3^, ^4^ In addition to the aforementioned clinical features, the 2q37 deletion syndrome has been reported to be associated with various other anomalies. Gastrointestinal anomalies such as pyloric stenosis as reported in reference,^5^ and central nervous system (CNS) anomalies such as holoprosencephaly^6^ have been observed in patients with this deletion syndrome. Additionally, nearly 20% of patients with 2q37 deletion syndrome have been reported to have cardiac anomalies such as ventricular septal defects and aortic.^1^ In this case, the patient was found to have a patent foramen ovale (PFO) which is consistent with the cardiac malformations associated with the 2q37 deletions.

## 2 CASE PRESENTATION

A 3‐year‐old female child was referred to the clinic for evaluation of easy fatigability, gross motor delay, low muscle tone, and flexible pes planus. The parents reported concerns that the child appeared to have difficulty keeping up with peers of the same age. The patient had no other reported medical issues and was growing and developing normally. Physical examination revealed no abnormalities on general examination, head, eyes, ears, nose, and throat examination, cardiovascular examination, and abdominal examination. Gait analysis showed a heel‐toe reciprocating gait with no deviations and normal muscle bulk. However, muscle tone was mildly reduced throughout. No tremors or other abnormal movements were noted (Tables 1 and 2).

**TABLE 1:** Vital signs of the presented case.

Vital signs	Value
Temperature	36.4°C
Height	38.1 inches (96.8 cm)
Weight	15.3 kg
BMI	16.37 kg/m^2^

Abbreviation: BMI, Body Mass Index.

**TABLE 2:** Laboratory results of the presented case.

Lab results	Value	Reference range
TSH	1.4	0.7–6.6 m(iu)/L
CK	69	<250 U/L
Calcium	10.7	8.8–10.8 mg/dL
Potassium	4.5	3.5–5 mmol/L
Creatinine	0.31	0.20–0.43 mg/dL

Abbreviations: CK, Creatine kinase; TSH, Thyroid‐stimulating hormone.

**FIGURE 1:**
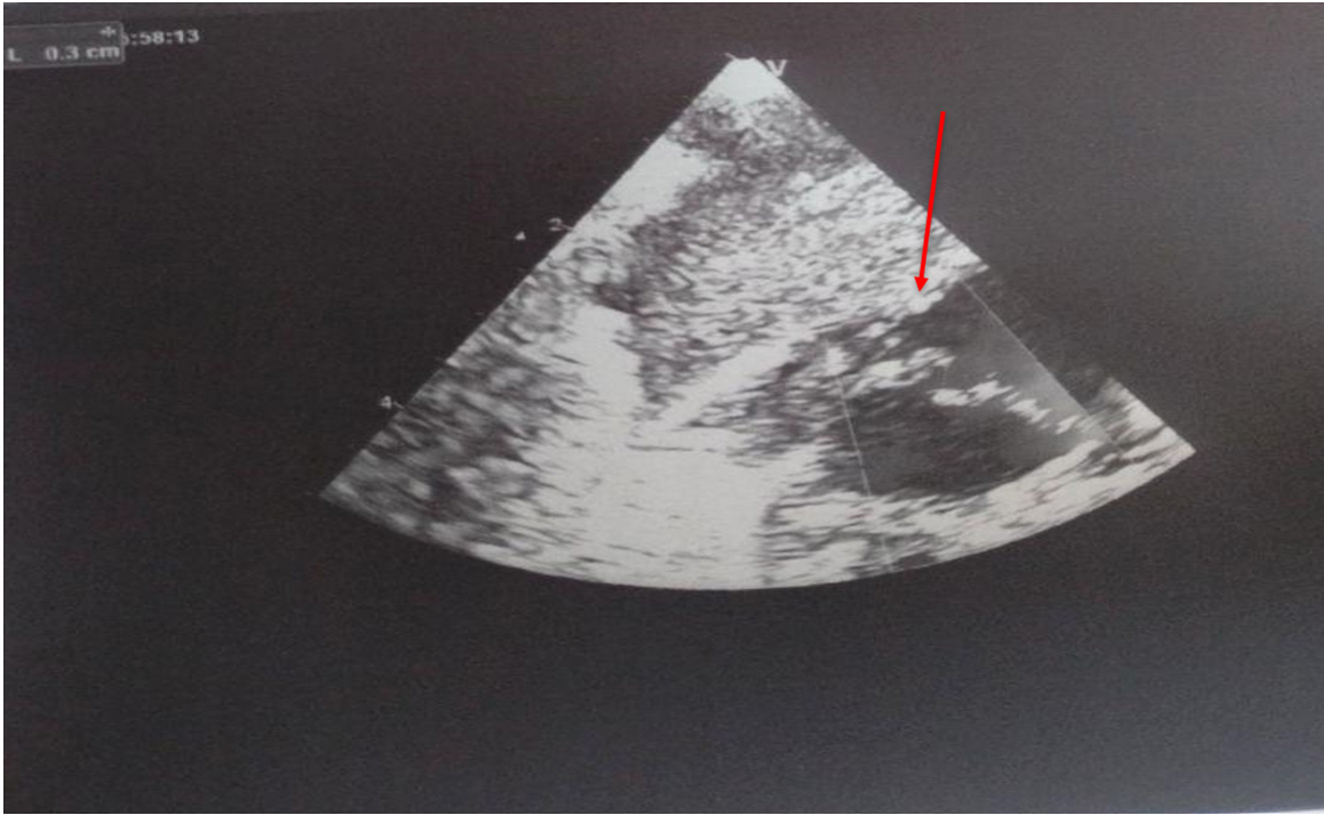
Echocardiography shows patent PFO. Levocardia, Situs solitus, normal intracardiac anatomy, patent foramen ovale (PFO) 3 mm with a left to right shunting, qualitatively normal right ventricular size, and systolic function, Normal left ventricular size and systolic function, and no evidence of significant pericardial effusion.

Further diagnostic testing including an echocardiogram revealed a patent foramen ovale (PFO) with left‐to‐right shunting (Figure 1). Genetic testing using comparative genomic hybridization (CGH) plus single nucleotide polymorphism (SNP) revealed a deletion of chromosome 2q37. The patient was advised to undergo two sessions of physical therapy per week and a follow‐up echocardiogram for her abnormal motor delay. After 1 year of physical therapy, there was improvement in hypotonia and gross motor delay.

## 3 DISCUSSION

The 2q37 deletion syndrome, first described in the literature in 1989, is a rare genetic disorder affecting approximately 74 individuals worldwide as reported in the medical literature to date. In 2004, US geneticist Dr. Kari Casas described 66 cases in addition to six case reports.^8^ The 2q37 deletion results in a range of clinical features, including developmental delay, brachydactyly of the third to fifth digits and/or toes, obesity, short stature, changes in facial appearance, and an autism spectrum disorder.^8^ Additionally, patients with this deletion syndrome also have some form of intellectual disability.^9^

The case presentation describes a 3‐year‐old female child who was referred to the clinic for evaluation of easy fatigability, gross motor delay, low muscle tone, and flexible pes planus. The parents reported concerns that the child appeared to have difficulty keeping up with peers of the same age. The patient had no other reported medical issues and was growing and developing normally.

Physical examination revealed no abnormalities on general examination, head, eyes, ears, nose, and throat examination, cardiovascular examination, and abdominal examination. However, gait analysis showed a heel‐toe reciprocating gait with no deviations and normal muscle bulk, but muscle tone was mildly reduced throughout. No tremors or other abnormal movements were noted.

Further diagnostic testing including an echocardiogram revealed a patent foramen ovale (PFO) with left‐to‐right shunting. PFO is a congenital heart defect that allows blood to flow between the right atrium and left atrium. This can lead to easy fatigability and low muscle tone. Genetic testing using comparative genomic hybridization (CGH) plus single nucleotide polymorphism (SNP) revealed a deletion of chromosome 2q37. Chromosome 2q37 deletion syndrome is a rare genetic disorder that can cause intellectual disability, developmental delay, and various physical abnormalities.

The patient was advised to undergo two sessions of physical therapy per week and a follow‐up echocardiogram for her abnormal motor delay. After 1 year of physical therapy, there was improvement in hypotonia and gross motor delay. The case highlights the importance of thorough physical examination and diagnostic testing in identifying underlying causes of developmental delays and physical abnormalities in children.

Patients with 2q37 deletion syndrome have been reported to have an increased prevalence of congenital heart defects, with approximately 20% of patients affected. Both septal defects and aortic coarctation have been described in these patients.^3^ In this case, the patient was found to have a patent foramen ovale in the atrial septum as confirmed by an echocardiogram and the ascending aorta, transverse arch, and descending aorta were found to be wide open and not blocking blood flow. The patient was growing and developing normally, both physically and mentally. Growth hormone deficiency has been reported in four patients with 2q37 gene mutations, which led to short stature, and all four patients experienced improved growth following growth hormone administration.^10^, ^11^, ^13^ The absence of major malformations in this patient can be attributed to the size of the deletion which was 2.63 MB and deletion size greater than 3 MB is usually associated with major malformations.^13^ Almost 30% of patients with 2q37 deletion syndrome have major malformations, including gastrointestinal, nervous system, and cardiac abnormalities as reported in.^3^

## 4 CONCLUSIONS

In conclusion, this case report presented a 3‐year‐old female child with easy fatigability, gross motor delay, low muscle tone, and flexible pes planus. The diagnosis was established through a combination of physical examination, echocardiogram, and genetic testing. The patient was found to have a patent foramen ovale (PFO) and a deletion of chromosome 2q37. Treatment of the patient with physical therapy improved her hypotonia and gross motor delay. The case highlights the importance of thorough physical examination and diagnostic testing in identifying underlying causes of developmental delays and physical abnormalities in children. Additionally, this case report provides an example of how multiple genetic and congenital disorders can coexist and influence clinical presentation.

## AUTHOR CONTRIBUTIONS

Ahmed Zaki, Nour Shaheen, Mohamed Hosny, Abdelraouf Ramadan, Abdulqadir J. Nashwan: Data Collection, Literature Search, Manuscript Preparation. All authors read and approved the final manuscript.

## FUNDING INFORMATION

This study was not funded.

## CONFLICT OF INTEREST STATEMENT

The authors declare that they have no competing interests.

## CONSENT

Written informed consent was obtained from the patient's legal guardian to publish this report in accordance with the journal's patient consent policy.

## ETHICAL STATEMENT

The article describes a case report. Therefore, no additional permission from our Ethics Committee was required.

## Data Availability

All data generated or analyzed during this study are included in this published article.
